# The Roles of Genetic and Early-Life Environmental Factors in the Association Between Overweight or Obesity and Hypertension: A Population-Based Twin Study

**DOI:** 10.3389/fendo.2021.743962

**Published:** 2021-10-05

**Authors:** Yu’e Xi, Wenjing Gao, Ke Zheng, Jun Lv, Canqing Yu, Shengfeng Wang, Tao Huang, Dianjianyi Sun, Chunxiao Liao, Yuanjie Pang, Zengchang Pang, Min Yu, Hua Wang, Xianping Wu, Zhong Dong, Fan Wu, Guohong Jiang, Xiaojie Wang, Yu Liu, Jian Deng, Lin Lu, Weihua Cao, Liming Li

**Affiliations:** ^1^ Department of Epidemiology and Biostatistics, School of Public Health, Peking University, Beijing, China; ^2^ Qingdao Municipal Center for Disease Control and Prevention , Qingdao, China; ^3^ Zhejiang Provincial Center for Disease Control and Prevention, Hangzhou, China; ^4^ Jiangsu Provincial Center for Disease Control and Prevention, Nanjing, China; ^5^ Sichuan Center for Disease Control and Prevention, Chengdu, China; ^6^ Beijing Center for Disease Prevention and Control, Beijing, China; ^7^ Shanghai Municipal Center for Disease Control and Prevention, Shanghai, China; ^8^ Tianjin Centers for Disease Control and Prevention, Tianjin, China; ^9^ Qinghai Center for Diseases Prevention and Control, Xining, China; ^10^ Heilongjiang Provincial Center for Disease Control and Prevention, Harbin, China; ^11^ Handan Center for Disease Control and Prevention, Handan, China; ^12^ Yunnan Center for Disease Control and Prevention, Kunming, China

**Keywords:** BMI, hypertension, genetics, early-life environments, twin study

## Abstract

**Aims/Hypothesis:**

We aimed to explore whether and to what extent overweight or obesity could increase the risk of hypertension, and further to estimate the roles of genetic and early-life familial environmental factors in their association.

**Methods:**

This prospective twin study was based on the Chinese National Twin Registry (CNTR), which collected information from self-report questionnaires. We conducted unmatched case-control analysis to examine the association between overweight or obesity and hypertension. And further to explore whether genetics and familiar environments shared within a twin pair, accounted for their association *via* co-twin matched case-control design. Generalized estimating equation (GEE) models and conditional logistic regressions were used in the unmatched and matched analyses, respectively. Then, we used logistic regressions to test the difference in odds ratios (ORs) between the unmatched and matched analyses. Finally, through bivariate twin model, the roles of genetic and environmental factors in the body mass index (BMI)- hypertension association were estimated.

**Results:**

Overall, we included a total of 30,617 twin individuals, of which 7533 (24.6%) twin participants were overweight or obesity and 757 (2.5%) developed hypertension during a median follow-up time of 4.4 years. In the GEE model, overweight or obesity was associated with a 94% increased risk of hypertension (OR=1.94, 95% confidence interval (CI): 1.64~2.30). In the conditional logistic regression, the multi-adjusted OR was 1.80 (95% CI: 1.18~2.74). The difference in OR between unmatched and matched analyses was significant (*P*=0.016). Specifically, overweight or obesity was not associated with hypertension risk in the co-twin design when we full controlled genetic and familiar environmental factors (OR=0.89, 95 CI: 0.46~1.72). After controlling for age and sex, we found the positive BMI-hypertension association was mainly explained by a genetic correlation between them (*r*_A_= 0.59, 95% CI: 0.44~1.00).

**Conclusions/Interpretation:**

Genetics and early-life environments shared by participants within a twin pair appear to account for the association between overweight or obesity and hypertension risk.

## Introduction

Raised blood pressure remains the leading cause of death globally, high systolic blood pressure accounted for 10·8 million deaths in 2019 (1). Unfortunately, hypertension has high prevalence but low rate of control. It is estimated that 1 in 4 men and 1 in 5 women (1.13 billion people), living with hypertension in 2015, but less than 1/5 have their blood pressure under control (2).

In the worldwide, a large number of people suffer from higher body mass index (BMI), including overweight and obesity. In 2016, a total of 39% adults are overweight, with a BMI ≥ 25 kg/m^2^, and 13% are obese (BMI ≥ 30 kg/m^2^) (3). Higher BMI is a major risk factor of hypertension. In the original Framingham cohort, Wilson et al. (4) found overweight and obese status were associated with increased risk of hypertension: the multi-adjusted risk ratios (RRs) among the overweight was 1.48 in men and 1.70 in women, while among the obese was 2.23 in men and 2.63 in women. In another Framingham study, weight loss led to a 21%~29% reduction in long-term hypertension risk (5). Mendelian randomization (MR) analysis, using genetic variants as the instrumental variable, has also demonstrated the causality between obesity and hypertension (6).

Genetic and early-life environmental factors, including shared fetal environment, childhood socioeconomic situation and adolescent environment, might have long-term effects on the subsequent risk of obesity (7–9) and hypertension (10–12). However, due to the limitations of general population-based study, their roles in the obesity-hypertension association are uncertain. Co-twin case-control analysis could address part of this difficulty, by controlling for genetic background and key shared familial environmental factors associated with obesity and hypertension. Twins are generally raised together, so they share their early-life environmental factors. They also share the same genetic predisposition and intrauterine environments. Therefore, as naturally matched pairs, co-twin case-control analyses provide an opportunity to explore the role of genetic and early-life environmental factors in the association between overweight or obesity and hypertension (13, 14).

The purpose of this study was to examine the association between overweight or obesity and hypertension, and to explore whether the association could be explained by genetic and common environmental factors shared within a twin pair, based on information from the Chinese National Twin Registry (CNTR).

## Methods

### Study Population

This prospective study enrolled twin participants from the CNTR (15), a twin population-based cohort study. Briefly, a total of 61,566 twin pairs, including 31,705 monozygotic (MZ) twins and 15,060 same-sex dizygotic (DZ) twins, from 11 provinces and cities in China were included at the baseline since 2001. Totally, the current study included 32,197 twins whose age was more than 18 years and participated in the resurvey.

**Figure 1:**
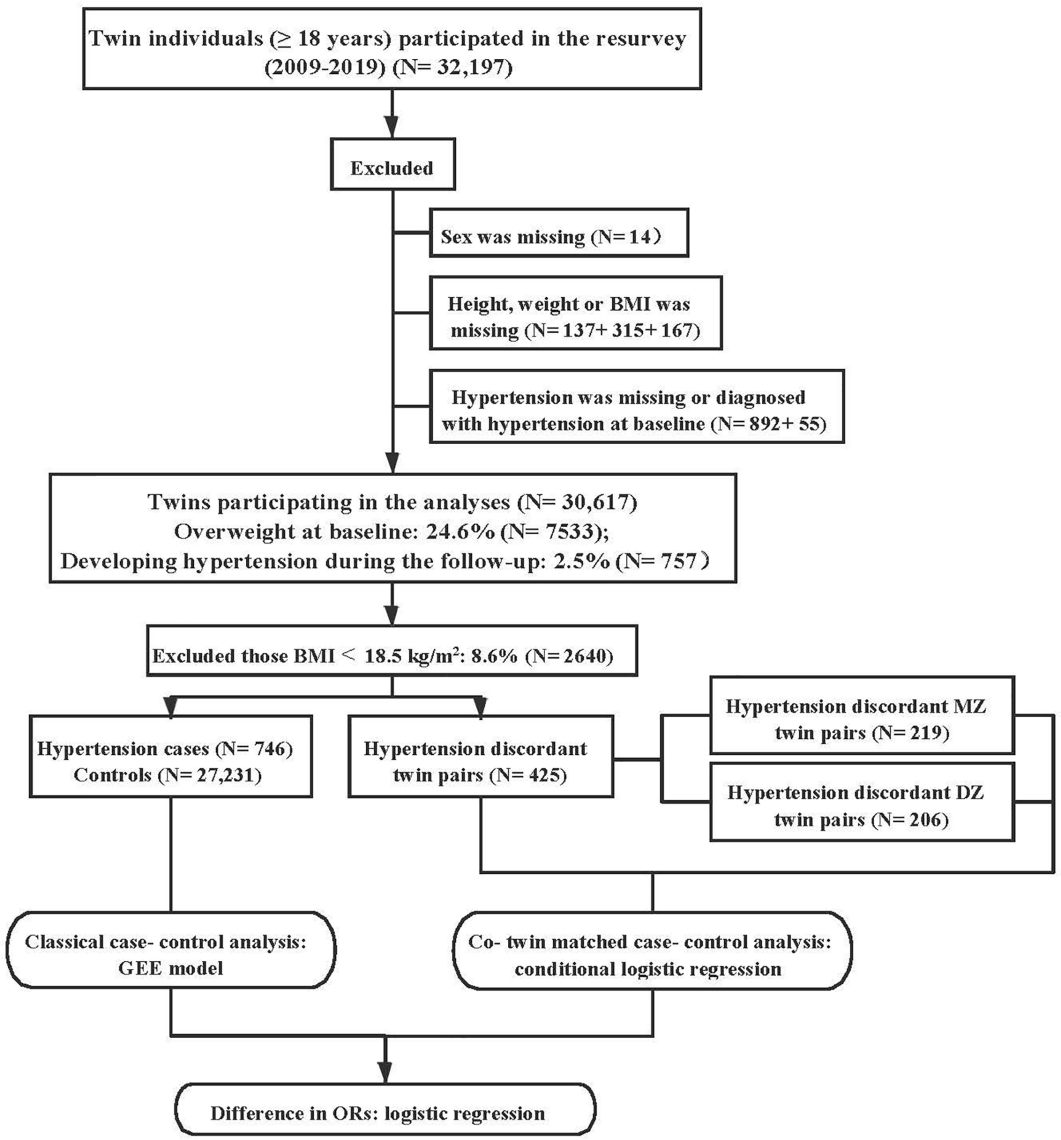
Flow chart of the study population and data analysis. BMI, body mass index; MZ, monozygotic; DZ, dizygotic; GEE, generalized estimating equation; OR, odds ratio.

We excluded 14 participants whose sex was missing. We also excluded 619 twins whose BMI was missing or with extreme outliers (under 3 or over 3-Z score of the BMI). In addition, we excluded those who were diagnosed with hypertension at baseline (892), and those who had no disease information at baseline or at the time of resurveys (55). Finally, a total of 30,617 twins were included for further association analysis (**Figure 1**).

We used the method of ‘Peas in the Pod Questionnaire (PPQ)’ to determine zygosity, asking about the degree of similarity shared by twins when they were at school age. Two former studies, based on the data from the CNTR, have verified its accuracy from 85% to 89% (16, 17).

All participants provided informed consent, and the study protocol was approved by the Ethics Committee at Peking University Health Science Center (IRB00001052-11029/14021).

### Data Collection

We collected information, including demographics (age, sex, marital status and educational attainment), lifestyles (smoking status, drinking status and physical activity), anthropometric measures (weight and height), twin zygosity and history of diseases (including diabetes and hypertension), from face-to-face questionnaire interview by trained interviewers.

### Ascertainment of Overweight or Obesity

Self-reported questionnaire was used to attain the information of height (in centimeter) and weight (in kilograms) at baseline. BMI was calculated by weight in kilograms divided by squared height in meters (kg/m^2^). According to the Chinese criteria of obesity (18), we categorized BMI into four groups: underweight (<18.5 kg/m^2^), normal weight (18.5 to 23.9 kg/m^2^), overweight (24.0 to 27.9 kg/m^2^) and obesity (≥ 28.0 kg/m^2^). In the current study, overweight or obesity was defined as BMI ≥24 kg/m^2^, that is, obesity was merged into overweight.

### Assessment of Hypertension

We collected the information on hypertension during the follow-up, asking “Have you ever been diagnosed with hypertension by a county/district level or above hospital”. Whether the participants are with hypertension or not depends on the doctor’s definite diagnosis, not just on their self-reported symptoms.

### Assessment of Covariates

Marital status was defined as married (or cohabitating) vs single (or divorced). Education attainment was categorized as primary, secondary and tertiary. Smoking status was grouped into never, former and current smoking. Drinking status was similarly divided into never, past and current drinking. Adequate physical activity was defined as exercising at least 30-minute moderate to high-intensity physical activity a day, and engaging in at least 5 days per week (19). Prevalence of diabetes was dichotomized into diabetes and diabetes-free.

### Statistical Analyses

#### Descriptive Statistics

The characteristics were compared between hypertension and non-hypertension groups. χ2 tests were used for categorical variables, independent sample t tests for continuous variables with normal distribution, and Mann Whitney U tests for continuous variables with non-normal distribution.

#### Case-Control Analyses

Generalized estimating equation (GEE) models were applied to assess the overweight-hypertension association, which are conceptually equivalent to logistic regressions for the classic case-control analysis, but controlling for the clustering of twins within a pair. In the co-twin matched case-control design, in which co-twin (both MZ and DZ twins) without hypertension was treated as a control for hypertension twin, we used conditional logistic regressions to explore the associations. Because cases and controls are matched for genetics and familial environments, discordant twin pairs were more informative than unrelated samples (20). Due to MZ twins share 100% genetic predisposition, while DZ twins share only 50%, we stratified the co-twin matched case-control analysis in MZ and DZ twins, respectively. Finally, logistic regressions were fitted to examine whether the ORs from the GEE model and conditional logistic regression are different, by comparing the distribution of overweight or obesity in the unmatched controls and co-twin controls (21). If a significant association between overweight or obesity and hypertension is only found in the GEE analysis, or OR in the co-twin analysis becomes significantly strengthened or attenuated, genetic and/or early-life environmental factors might play roles in their association (22, 23). In contrast, if difference in ORs between the GEE model and conditional logistic regression is not significant, then the genetic and shared familiar factors might not account for the observed association (14, 21, 24).

Age, sex, marital status, education attainment, smoking status, drinking status, physical activity and diabetes were considered as potential confounders. The basic-adjusted models were controlled for age and sex (twins within a pair have the same age, thus only sex was adjusted in the co-twin analysis). The multi-adjusted models were further adjusted for marital status, education, smoking, drinking, physical activity and diabetes.

#### Twin Modeling

The classical twin method decomposes the phenotype variation, based on the phenotypic correlations between twin pairs: MZ twins share 100% genetic materials, whereas DZ twins average share 50% of their segregating genes; all twins are correlated for environmental influences to the same extent. In this study, the liability threshold model, an extension of the classical twin modelling was used, assuming individual differences in a trait come from additive genetic (A), nonadditive genetic (D), shared environmental (C) and nonshared environmental (E) influences. The bivariate genetic model estimates the influence of A, C, D and E on each trait (BMI and hypertension), and also explores how much of the association (phenotypic correlation, *r*_ph_) between them can be partitioned into addictive genetic (*r*_A_), nonaddictive genetic (*r*_D_), shared environmental (*r*_C_), and unique environmental (*r*_E_) correlation. Because of the effects of C and D are confounded in the classical twin model, including twin pairs reared together, they cannot be calculated simultaneously (25). For both BMI and hypertension, the ADE model was only fitted when the intraclass correlation coefficient (ICC) of MZ was more than double that of DZ twins. To investigate how much of the BMI-hypertension association is attributed to genetic or environmental correlations, we compared the difference of cross-trait, cross-twin correlations (CTCTs) between MZ and DZ twin pairs. A higher CTCT in MZ than in DZ twin pairs indicates that BMI and hypertension are associated because of correlated genetic influences. Based on the full ACE models, several nested models, including AE, CE and E, were fitted by dropping C, A and both components for the selection of best fitting model.

The likelihood ratio test was used to assess the fit of nested models, which approximately follows a χ2 distribution in that the degree of freedom is equal to the difference of the parameters number between the two models. Each nested model was compared with the full model to choose the best fitting model. The Akaike’s information criterion (AIC) was applied for the model selection, in which lower values suggesting a better balance between explanatory power and parsimony (26).

Data cleaning and statistical analyses were performed using Stata/MP 14.0. Structural equation models were fitted in R 3.5.1 with the use of an open source software package named OpenMx (version 2.14.11) (27).

## Results

### Characteristics of the Study Population

Overall, a total of 30,617 twin individuals, including 17,571 (57.4%) men and 13,570 (44.8%) DZ twins were included in the current study. The average age at baseline was 32.6 ± 11.4 years, the median follow-up time was 4.4 years. 7533 participants (24.6%) were overweight, and 757 (2.5%) participants were diagnosed with hypertension in the resurvey. And among the overweight or obesity individuals at baseline, 371 (4.9%) twins developed hypertension during the follow-up. Participants who had hypertension were more likely to be older, male, overweight, current smokers, current drinkers, to have adequate physical activity, higher education, and to be diagnosed with diabetes, compared with those who were hypertension-free (**Table 1**).

**Table 1:** Characteristics of the study participants by hypertension diagnosis (N = 30617).

Characteristics	Hypertension-free (N = 29860)	Hypertension (N = 757)	Total (N = 30617)	*P* value
Age, years (mean, SD)	32.2 (11.2)	47.0 (11.3)	32.6 (11.4)	<0.001
Male, n (%)	17008 (57.0)	563 (74.4)	17571 (57.4)	<0.001
DZ, n (%)	13261 (44.9)	309 (41.1)	13570 (44.8)	0.039
Follow-up time, median (IQR)	4.4 (2.0, 5.9)	5.3 (4.4, 6.0)	4.4 (2.1, 5.9)	<0.001
BMI, kg/m^2^ (%)				<0.001
Normal weight (BMI, 18.5–23.9)	20069 (67.2)	375 (49.5)	20444 (66.8)	
Underweight (BMI < 18.5)	2629 (8.8)	11 (1.5)	2640 (8.6)	
Overweight (BMI ≥ 24)	7162 (24.0)	371 (49.0)	7533 (24.6)	
Married, n (%)	16584 (76.7)	664 (91.1)	17248 (77.2)	<0.001
Educational attainment, n (%)				<0.001
Primary	2717 (12.6)	180 (24.7)	2897 (13.0)	
Secondary	12541 (58.0)	477 (65.3)	13018 (58.2)	
Tertiary	6366 (29.4)	73 (10.0)	6439 (28.8)	
Smoking status, n (%)				<0.001
Never	15384 (71.2)	444 (60.8)	15828 (70.8)	
Current	5957 (27.6)	268 (36.7)	6225 (27.9)	
Former	279 (1.3)	18 (2.5)	297 (1.3)	
Drinking status, n (%)				<0.001
Never	17092 (79.1)	492 (67.6)	17584 (78.7)	
Current	4362 (20.2)	226 (31.0)	4588 (20.5)	
Former	159 (0.7)	10 (1.4)	169 (0.8)	
Adequate physical activity, n (%)	8568 (42.6)	354 (53.7)	8922 (42.9)	<0.001
Diabetes, n (%)	178 (0.6)	17 (2.2)	195 (0.6)	<0.001

BMI, body mass index; DZ, dizygotic; SD, standard deviation; IQR, interquartile range.

### Case-Control Analyses

After adjustment of age, sex, marital status, education, smoking, drinking, physical activity and diabetes, overweight or obesity increased a 94% risk of hypertension in the GEE model (OR=1.94, 95% CI: 1.64~2.30) (**Table 2**). In the multi-adjusted conditional logistic regression, overweight or obesity was associated with an 80% higher hypertension risk (OR=1.80, 95% CI: 1.18~2.74). In the matched analysis of DZ twins, controlling 50% genetic factors, overweight or obesity increased the risk of hypertension under the control of confounders (OR=2.86, 95% CI: 1.57~5.21). However, in the co-twin case-control analysis of MZ twins, the association between overweight or obesity and hypertension was not significant, the multi-adjusted OR was 0.89 (95% CI: 0.46~1.72) (**Table 3**). When we adjusted for the potential confounding variables, the difference in ORs between unmatched and matched case-control analyses was significant in all twin pairs (*P*=0.016), suggesting genetic, early-life familial environmental factors or both of them may partially contribute to the overweight-hypertension association (**Table 4**).

**Table 2:** ORs (95% CIs) of overweight or obesity-hypertension association (normal BMI as the reference) from the GEE models.

Models	No. of cases	OR (95% CI)
Model^a^	27977	2.03 (1.73,2.39)
Model^b^	20888	1.91 (1.62,2.25)
Model^c^	19394	1.94 (1.64,2.30)

BMI, body mass index; GEE, generalized estimating equation; OR, odds ratio; CI, confidence interval.

a Adjusted for age and sex.

b Adjusted for age, sex, marital status and education.

c Adjusted for age, sex, marital status, education, smoking status, alcohol consumption, physical activity and diabetes.

**Table 3:** ORs (95% CIs) for the association between overweight or obesity and hypertension in co-twin control analyses using hypertension discordant twin pairs from the conditional logistic regressions.

Co-twin without hypertension	Twin with hypertension					
	MZ+DZ		DZ		MZ	
	Normal BMI	Overweight	Normal BMI	Overweight	Normal BMI	Overweight
Normal BMI	175	77	72	56	103	21
Overweight	45	128	22	56	23	72
OR (95% CI)^a^	1.63 (1.12,2.37)		2.39 (1.45,3.95)		0.91 (0.51,1.65)	
OR (95% CI)^b^	1.60 (1.09,2.33)		2.44 (1.46,4.09)		0.84 (0.45,1.54)	
OR (95% CI)^c^	1.80 (1.18,2.74)		2.86 (1.57,5.21)		0.89 (0.46,1.72)	

BMI, body mass index; MZ, monozygotic; DZ, dizygotic; OR, odds ratio; CI, confidence interval.

The 425 (206 DZ and 219 MZ) hypertension discordant pairs were divided into four groups with respect to exposure (overweight) status. In 175 (72 DZ and 103 MZ) twin pairs, both had normal BMI. In 128 (56 DZ and 72 MZ) twin pairs, both were overweight. In 77 (56 MZ and 21 MZ) twin pairs, the healthy (hypertension-free) co-twin had normal weight and the diseased twin was overweight. In 45 (22 DZ and 23 MZ) twin pairs, the diseased co-twin had normal BMI and the healthy twin was overweight.

a Adjusted for sex.

b Adjusted for sex, marital status, education.

c Adjusted for sex, marital status, education, smoking status, alcohol consumption, physical activity and diabetes.

**Table 4:** Differences in ORs (95% CIs) for the unmatched GEE models and matched co-twin control analyses (the difference in overweight or obesity between unmatched and co-twin matched controls).

Models	MZ+DZ			DZ			MZ		
	No. of cases	OR (95% CI)	*P* value	No. of cases	OR (95% CI)	*P* value	No. of cases	OR (95% CI)	*P* value
Model^a^	27656	1.26 (1.03,1.55)	0.023	27437	1.22 (0.91,1.63)	0.190	27450	1.30 (0.99,1.72)	0.062
Model^b^	20574	1.32 (1.07,1.62)	0.009	20369	1.26 (0.94,1.69)	0.122	20375	1.36 (1.03,1.81)	0.032
Model^c^	19115	1.30 (1.05,1.62)	0.016	18924	1.26 (0.92,1.72)	0.146	18936	1.34 (1.00,1.79)	0.052

MZ, monozygotic; DZ, dizygotic; GEE, generalized estimating equation; OR, odds ratio; CI, confidence interval.

a Adjusted for age and sex.

b Adjusted for age, sex, marital status and education.

c Adjusted for age, sex, marital status, education, smoking status, alcohol consumption, physical activity and diabetes.

### Twin Model Fitting

For both BMI and hypertension, the ICCs of MZ twins were larger than DZ twins, which implied genetic influences on both traits. Compared with DZ twins (*r*=0.10, 95% CI: 0.04~0.16), the CTCT was higher for MZ twins (*r*=0.18, 95% CI: 0.13~0.23), suggesting a genetic correlation between BMI and hypertension (**Table 5**). The analysis revealed that the full ACE model was the best-fitting one, which was therefore used for estimation of genetic and environmental influences (**Supplementary Table 3**). The additive genetic factors explained 45% (95% CI: 41%~49%) and 32% (95% CI: 8%~59%) variance of BMI and hypertension, respectively. The genetic correlation, which pointed to what degree genetic variance of BMI predicted the genetic influences on hypertension, was 0.59 (95% CI: 0.44~1.00). The shared and non-shared environmental correlations were not significant (**Table 6**).

**Table 5:** Correlations (95% CIs) for BMI, hypertension, and BMI-hypertension by zygosity.

Zygosity	Within-trait, cross-twin		Cross-trait, within-twin	Cross-trait, cross-twin
	BMI	Hypertension		
MZ	0.70 (0.68,0.72)	0.73 (0.67,0.79)	0.19 (0.09,0.24)	0.18 (0.13,0.23)
DZ	0.51 (0.48,0.53)	0.57 (0.45,0.67)	0.22 (0.16,0.28)	0.10 (0.04,0.16)

BMI, body mass index; MZ, monozygotic; DZ, dizygotic; CI, confidence interval.

**Table 6:** Parameter estimates (95% CIs) from the best-fitting bivariate ACE full model of BMI and hypertension.

	Variance components		Correlation	
	BMI	Hypertension	
			*r*_Ph_	0.21 (0.17,0.25)
A	0.45 (0.41,0.49)	0.32 (0.08,0.59)	*r*_A_	0.59 (0.44,1.00)
C	0.36 (0.32,0.40)	0.41 (0.15,0.63)	*r*_C_	-0.06 (-1.00,1.00)
E	0.19 (0.19,0.20)	0.27 (0.21,0.33)	*r*_E_	0.05 (-0.05,0.14)

BMI, body mass index; CI, confidence interval; A, Additive genetic factors; C, shared environmental factors; E, non-shared environmental factors; r_Ph_, phenotypic correlation; r_A_, genetic correlation; r_C_, shared environmental correlation; r_E_, non-shared environmental correlation.

### Supplementary Analysis

Considering the different effects of overweight and obesity on the risk of incident hypertension, we also separated participants who were overweight (BMI: 24- 28 kg/m2) and obesity (BMI ≥ 28 kg/m2) to explore their associations with hypertension. Under the control of potential influenced factors, both overweight and obesity increased hypertension risk, the ORs were 1.87 (95% CI: 1.57~2.24) and 2.64 (95% CI: 1.92~3.61), respectively (**Supplementary Table 1**). In the matched case-control study, overweight was associated with a 72% increased hypertension risk (OR=1.72, 95% CI: 1.12~2.67). However, due to the limited sample size, we found a borderline significant obesity-hypertension association in the matched analyses (OR=9.26, 95% CI: 1.00~85.50) (**Supplementary Table 2**). The different in ORs between unmatched and matched designs was significant for the obesity (*P*=0.009), but not significant for the overweight (*P*=0.075) (**Supplementary Table 3**).

## Discussion

In this large-scale, nationwide Chinese twin study, we found that both overweight and obesity were significantly associated with increased hypertension risk. And the associations were different between unmatched and matched analyses, and even non-significant in the co-twin analysis of MZ twins, indicating genetic or both genetic and environmental factors shared between co-twins are likely to contribute to the association. Furthermore, we found a positive correlation between BMI and hypertension, which was explained by a genetic correlation, providing evidence for the contribution of overlap genetic factors on their association.

Consistent with our findings, a growing number of studies have reported that overweight and obesity were independently associated with increased risk of hypertension. Using 5209 participants aged 30 to 62 years from the original Framingham cohort, Wilson et al. (4) found overweight and obese status were positively associated with hypertension: compared with those with normal weight, overweight increased a 48% (95% CI: 1.24~1.75) and 70% (95% CI: 1.48~1.94) risk of hypertension in the men and women, respectively; the age-adjusted RRs were 2.23 (95% CI: 1.75~2.84), and 2.63 (95% CI: 2.20~3.15) in male and female obese individuals, respectively. After 6.38-year follow-up, Qi et al. (28) reported a positive association between BMI and hypertension in the Chines: the RR was 3.13 (95% CI: 2.84~3.45) for the obesity. In the Framingham Study, Moore et al. (5) found weight loss ≥ 6.8 kg led to a 28% (RR=0.72, 95% CI: 0.49~1.05) and a 37% (RR=0.63, 95% CI: 0.42~0.95) reduction in hypertension risk for middle-aged and older adults, respectively; And sustained weight loss also reduced the hypertension risk: 22% and 26% for middle-aged and older adults, respectively. These studies provided evidence that overweight and obesity were independently associated with hypertension risk.

The mechanisms underlying the overweight (obesity)-hypertension association are complex and not completely understood. Obesity can directly produce a variety of structural and functional changes of the cardiovascular system, including lower cardiac output, poorer left ventricular systolic function, higher peripheral resistance, increased left ventricular mass, left ventricular wall thickness and internal dimension (29). In addition, obesity is associated with mechanisms that could increase sympathetic nervous system (SNS) activity, which is believed to play an important role in the development of hypertension. Angiotensin II could increase SNS activity, while angiotensinogen is expressed in visceral adipocytes (30, 31). In the obesity, the inhibitory of arterial baroreflex on SNS activity is reduced, contributing to the increased SNS activity to muscle and kidney (32, 33). And the dysregulation of the hypothalamic-pituitary-adrenal axis, characterized by obesity, seems to be important to the activation of the SNS in obese humans (34).

The co-twin case-control analysis could explore associations, under the control of genetic and unmeasured early-life environmental factors. In the current study, we found the overweight-hypertension association was attenuated in the matched study, and even disappeared in the co-twin analysis of MZ twins, indicating that the observed association was fully explained by genetic and familiar factors shared within a twin pair. Our results indicate these with family history of obesity may have high hypertension risk, which showed the important to prevent hypertension in these population. However, not consistent with our findings, MR analysis has demonstrated the causal effect of obesity on hypertension. Including 119,859 participants from UK Biobank, Lee et al. (6) showed a positive association between genetically instrumented higher BMI and hypertension risk (OR= 1.64, 95% CI: 1.48~1.83). In a Korea cohort study, using genetic risk scores (GRS), created by 6 single-nucleotide polymorphisms associated with BMI, researchers found a causal effect of BMI on hypertension (OR: 1.13~1.26) (35). In addition, a large number of studies have investigated the biological mechanisms underlying obesity-hypertension association. Therefore, our findings based on statistics analysis should be interpreted with caution. More large studies from Chinese population are warranted to verify our results.

Furthermore, using bivariate twin model, we further found the positive BMI-hypertension association was explained by a genetic correlation between them, providing evidence for the contribution of overlap genes on their relationship. Including a total of 913 subjects from 179 families, Li et al. (36) found waist circumference (WC) was genetically correlated with systolic blood pressure (*r*_A_=0.27), but found no significant genetic correlations between BMI and blood pressure. Although we found no study exploring the genetic link between overweight (obesity) and hypertension, numerous studies provide evidences for the pleiotropy between obesity and hypertension. *FTO* (fat mass and obesity-associated) gene, the first identified gene for obesity, is the strongest BMI related genetic factors (37, 38). Meta-analysis has demonstrated the associations between *FTO* SNPs and obesity risk (39, 40). It is reported that *FTO* gene is highly expressed in the hypothalamic nuclei (41, 42), involving in the control of energy homeostasis (43) and regulation of blood pressure (44). To date, several studies have investigated the association between *FTO* variants and risk of hypertension. Although the results are inconsistent, many large studies showed some of *FTO* genotypes significantly increased risks of hypertension (45). Many other genes, such as *MC4R* (46–48), TNF-α (49–51), *LEP* (*LEPR*) (52–56) and *β2AR* (57, 58), were all associated with both obesity and hypertension. However, because of the lack of comparable co-twin studies, the roles of shared genetic factors in the relationship between overweight or obesity and hypertension are still needed to be explored.

Although our co-twin analysis reported that early-life environment might contribute to the overweight-hypertension association, the common environmental correlation was not significant in the twin model. Not consistent with our results, as important parts of early-life environments, a growing body of studies described that poor fetal and early postnatal growth were associated with subsequent risk of obesity and hypertension. The majority of epidemiological studies utilize birth weight and gestational age as proxy markers for suboptimal utero growth. Abundant evidences suggested that high birth weight (HBW) and large for gestational age were associated with an increased risk of obesity later in life (59–61). In a meta-analysis, including 14 cohort studies, HBW (≥4000g) was associated with higher risk of obesity (OR=1.43, 95% CI: 1.25~1.64), but not low birth weight (LBW) (<2500 g) (60). Furthermore, low birth weight (LBW) was associated with higher hypertension risk (62–65). In the Shanghai Women’s Health Study and the Shanghai Men’s Health Study, an excess risk of hypertension was observed for LBW, hazard ratio (HR) was 1.20 (95% CI: 1.11~1.30) (63). In the Swedish twin study on the fetal origins of hypertension, Bergvall et al. (65) provided evidence that LBW was associated with increased hypertension risk. Although early-life environmental factors are associated with both obesity and hypertension, whether those factors confound the overweight (obesity)-hypertension association is unknown.

Some limitations of this study need to be mentioned. First, we calculated BMI from self-reported height and weight, which could have led to an underestimation of the overweight. Besides, we collected disease information from self-reported questionnaires without measuring the blood pressure. Because of the higher proportion of patients who were not aware of their hypertension (12.3%~24.7%) in the Chinese adults (66, 67), individuals with undiagnosed hypertension might have been misclassified as hypertension-free. Both of those could have led to biased estimation for the overweight-hypertension association. Finally, despite the large sample sizes, the number of cases for stratification analyses was small, especially for the co-twin control analysis. Specifically, we obtained a borderline significant association between obesity and hypertension, due to the limited sample sizes. Thus, further investigations are needed to assess whether the genetics and early-life environmental factors account for this association.

Nonetheless, the current study has several strengths. First, the large nationwide twin cohort allowed us to explore the effects of overweight and obesity on hypertension, and simultaneously estimate the potential influence of genetic and early-life environmental factors in their relationship with sufficient power. We used GEE models, controlling for the clustering of twins within a pair, to provide evidence for the overweight-hypertension association. And further assessing the roles of genetic and familiar factors on the given association, *via* co-twin case-control design. Second, we only included twins who were diagnosed with hypertension during the follow-up, making the temporality clear and thus minimize the possibility of reverse causality. Third, due to DZ twins only share 50% of their genetic predisposition, co-twin matched case-control analyses including both MZ and DZ twins do not completely control for genetic factors. Therefore, we further repeated co-twin design in MZ and DZ twins separately, which verified our results.

In conclusion, with the current study, we add evidence to the positive link between overweight (obesity) and hypertension, and show the importance of genetic and family environmental factors for their association. That is, due to the common genetic predisposition, individuals with higher BMI seem to be more likely to develop into hypertension. A next step would be to verify our findings in more prospective studies, and find more genes and environments responsible for the overweight (obesity)-hypertension association.

## Data Availability Statement

The original contributions presented in the study are included in the article/**Supplementary Material**. Further inquiries can be directed to the corresponding authors.

## Ethics Statement

The studies involving human participants were reviewed and approved by the Ethics Committee at Peking University Health Science Center (IRB00001052-11029/14021). The patients/participants provided their written informed consent to participate in this study.

## Author Contributions

WC and WG contributed to the study design and supervise the whole project. ZP, MY, HW, XPW, ZD, FW, GJ, XJW, YL, JD, and LL contributed to conduct field study and collect the data. KZ, JL, CY, SW, TH, DS, CL, and YP contributed to the results interpretation and provided critical comments. LML helped to design and supervise the whole study and obtain funding. YX analyzed data and drafted the manuscript. All authors contributed to the article and approved the submitted version.

## Funding

This study was funded by National Natural Science Foundation of China (82073633, 81973126, 81711530051, 81573223, 81473041), and Special Fund for Health Scientific Research in the Public Welfare (201502006, 201002007). The funders had no role in study design and conduct, data collection, analysis and interpretation, preparation of the manuscript, or the decision to publication.

## Conflict of Interest

The authors declare that the research was conducted in the absence of any commercial or financial relationships that could be construed as a potential conflict of interest.

The reviewer XT declared a shared affiliation, with no collaboration, with ZP to the handling editor at the time of review.

## Publisher’s Note

All claims expressed in this article are solely those of the authors and do not necessarily represent those of their affiliated organizations, or those of the publisher, the editors and the reviewers. Any product that may be evaluated in this article, or claim that may be made by its manufacturer, is not guaranteed or endorsed by the publisher.
