# Supplementary material for: The Roles of Genetic and Early-Life Environmental Factors in the Association Between Overweight or Obesity and Hypertension: A Population-Based Twin Study
Source: Front Endocrinol (Lausanne). 2021 Oct 5;12:743962. doi: 10.3389/fendo.2021.743962 (PMC8525506; doi:10.3389/fendo.2021.743962)
Supplement: Supplementary file 1 [file DataSheet_1.doc]

**Supplementary Material**

**Questionnaires**

1. **What is the highest level of school education you ever received?**

No formal school

Primary school

Middle school

High school

Technical school/college

University

1. **What is your current marital status?**

Married

Separated/divorced

Widowed

Never married

1. **Are you a smoker?**

Never smoker

Smoker

Ex regular smoker

1. **Are you a drinker?**

Never drinker

Drinker

Ex regular drinker

1. **Do you engage in at least 30-minute moderate to high-intensity physical activity per day, and at least five days per week?**

No

Yes

Unclear

1. **Have you ever been diagnosed with diabetes by a county/district level or above hospital?**

No

Yes

Supplementary Table 1. Odds ratios (ORs) and 95% confidence intervals (CIs) of overweight and obesity in relation to hypertension from the Generalized Estimating Equation models among stratified analysis

| BMI | Models | No. of cases | OR (95% CI) |
| --- | --- | --- | --- |
| Overweight | Model^a^ | 26,930 | 1.95 (1.65,2.32) |
|  | Model^b^ | 19,973 | 1.85 (1.56,2.20) |
|  | Model^c^ | 18,525 | 1.87 (1.57,2.24) |
| Obesity | Model^a^ | 21,491 | 2.73 (2,02,3.69) |
|  | Model^b^ | 15,407 | 2.50 (1.84,3.39) |
|  | Model^c^ | 14,281 | 2.64 (1.92,3.61) |

*Abbreviations*: BMI, body mass index.

^a^Adjusted for age and sex.

^b^Adjusted for age, sex, marital status and education.

^c^Adjusted for age, sex, marital status, education, smoking status, alcohol consumption, physical activity and diabetes.

Supplementary Table 2 ORs (95% CIs) for the association between overweight (obesity) and hypertension in co-twin control analyses using hypertension discordant twin pairs from the conditional logistic regressions

| Co-twin without hypertension | Twin with hypertension | | | | |
| --- | --- | --- | --- | --- | --- |
|  | Overweight | |  | Obesity | |
|  | Normal BMI | Overweight |  | Normal BMI | Obesity |
| Normal BMI | 175 | 70 |  | 175 | 7 |
| Overweight (Obesity) | 43 | 91 |  | 2 | 16 |
| OR (95% CI)^a^ | 1.58 (1.08,2.32) | |  | 3.27 (0.67,15.99) | |
| OR (95% CI)^b^ | 1.55 (1.05,2.30) | |  | 3.60 (0.71,18.23) | |
| OR (95% CI)^c^ | 1.72 (1.12,2.67) | |  | 9.26 (1.00,85.50) | |

*Abbreviations*: BMI, body mass index; OR, odds ratio; CI, confidence interval..The hypertension discordant pairs (379 for overweight, and 200 for obesity) were divided into four groups with respect to exposure (overweight, obesity) status. In 175 twin pairs, both were normal BMI. In 91(16) twin pairs, both were overweight (obesity). In 70 (7) twin pairs, the healthy (hypertension-free) co-twin had normal weight and the diseased twin was overweight (obesity). In 43 (2) twin pairs, the diseased co-twin had normal BMI and the healthy twin was overweight (obesity).

^a^Adjusted for sex.

^b^Adjusted for sex, marital status, education.

^c^Adjusted for sex, marital status, education, smoking status, alcohol consumption, physical activity and diabetes.

Supplementary Table 3 Differences in ORs (95% CIs) for the unmatched Generalized estimating equation (GEE) models and matched co-twin control analyses (the difference in overweight and obesity between unmatched and co-twin matched controls)

| Models | Overweight | | |  | Obesity | | |
| --- | --- | --- | --- | --- | --- | --- | --- |
|  | No. of cases | OR (95% CI) | *P* value |  | No. of cases | OR (95% CI) | *P* value |
| Model^a^ | 26,642 | 1.21 (0.97,1.49) | 0.086 |  | 21,335 | 1.55 (1.04,2.33) | 0.033 |
| Model^b^ | 19,691 | 1.26 (1.01,1.56) | 0.039 |  | 15,254 | 1.63 (1.08,2.46) | 0.020 |
| Model^c^ | 18,276 | 1.23 (0.98,1.54) | 0.075 |  | 14,146 | 1.76 (1.16,2.69) | 0.009 |

*Abbreviations*: OR, odds ratio; CI, confidence interval. ^a^Adjusted for age and sex.

^b^Adjusted for age, sex, marital status and education.

^c^Adjusted for age, sex, marital status, education, smoking status, alcohol consumption, physical activity and diabetes.

Supplementary Table 4. Model fitting results of bivariate analyses of BMI and hypertension

| Models | Estimated variables | -2LL | df | AIC | ΔLL | Δdf | *P* value |
| --- | --- | --- | --- | --- | --- | --- | --- |
| Step 1: saturated model |  |  |  |  |  |  |  |
| 1) Full saturated model | 28 | 57203.52 | 46708 | -36212.48 |  |  |  |
| 2) βa covariate dropped | 24 | 59579.28 | 46712 | -33844.72 | 2375.76 | 4 | <0.001 |
| 3) Means constrained across twin birth order for BMI | 26 | 57203.52 | 46710 | -36216.48 | 0.00 | 2 | 1.000 |
| 4) Means constrained across twin zygosity for BMI | 25 | 57214.49 | 46711 | -36207.51 | 10.96 | 3 | 0.012 |
| 5) Variances constrained across twin birth order for BMI | 24 | 57204.64 | 46712 | -36219.36 | 1.11 | 4 | 0.892 |
| 6) Variances constrained across twin zygosity for BMI | 23 | 57205.97 | 46713 | -36220.03 | 2.45 | 5 | 0.785 |
| 7) Thresholds constrained across twin birth order for hypertension | 21 | 57210.67 | 46715 | -36219.33 | 7.14 | 7 | 0.414 |
| 8) Thresholds constrained across twin zygosity for hypertension | 20 | 57212.61 | 46716 | -36219.39 | 9.08 | 8 | 0.335 |
| **9) Covariances constrained across twin zygosity ^a^** | **16** | **57214.65** | **46720** | **-36225.35** | **11.12** | **12** | **0.518** |
| Step 2: ACE model |  |  |  |  |  |  |  |
| **10) Full ACE model ^a^** | **16** | **57215.31** | **46721** | **-36226.69** | **11.79** | **13** | **0.545 ^b^** |
| 11) AE model | 13 | 57448.36 | 46724 | -35999.64 | 233.05 | 3 | <0.001 |
| 12) CE model | 13 | 58035.85 | 46724 | -35412.15 | 820.54 | 3 | <0.001 |
| 13) E model | 10 | 67583.07 | 46727 | -25870.93 | 10367.75 | 6 | <0.001 |

*Abbreviations*: BMI, body mass index; A, additive genetic factors; C, shared environmental factors; E, unique environmental factors; LL, log likelihood; df, degrees of freedom; AIC, Akaike’s Information Criterion; β, the effect of age and sex. ^a^ Best-fitting model, ^c^ Comparing with full saturated model.
