# Supplementary material for: The Roles of Genetic and Early-Life Environmental Factors in the Association Between Overweight or Obesity and Hypertension: A Population-Based Twin Study
Source: Front Endocrinol (Lausanne). 2021 Oct 5;12:743962. doi: 10.3389/fendo.2021.743962 (PMC8525506; doi:10.3389/fendo.2021.743962)
Supplement: Supplementary file 2 [file DataSheet_2.docx]

Table 1. Characteristics of the study participants by hypertension diagnosis (N = 30617)

| Characteristics | Hypertension-free (N=29860) | Hypertension (N=757) | Total (N=30617) | *P* value |
| --- | --- | --- | --- | --- |
| Age, years (mean, SD) | 32.2 (11.2) | 47.0 (11.3) | 32.6 (11.4) | <0.001 |
| Sex(male), n (%) | 17008 (57.0) | 563 (74.4) | 17571 (57.4) | <0.001 |
| DZ, n (%) | 13261 (44.9) | 309 (41.1) | 13570 (44.8) | 0.039 |
| Follow-up time, median (IQR) | 4.4 (2.0, 5.9) | 5.3 (4.4, 6.0) | 4.4 (2.1, 5.9) | <0.001 |
| BMI, kg/m^2^ (%) |  |  |  | <0.001 |
| Normal weight (BMI, 18.5–23.9) | 20069 (67.2) | 375 (49.5) | 20444 (66.8) |  |
| Underweight (BMI < 18.5) | 2629 (8.8) | 11 (1.5) | 2640 (8.6) |  |
| Overweight (BMI ≥ 24) | 7162 (24.0) | 371 (49.0) | 7533 (24.6) |  |
| Marital status (Married), n (%) | 16584 (76.7) | 664 (91.1) | 17248 (77.2) | <0.001 |
| Educational attainment, n (%) |  |  |  | <0.001 |
| Primary | 2717 (12.6) | 180 (24.7) | 2897 (13.0) |  |
| Secondary | 12541 (58.0) | 477 (65.3) | 13018 (58.2) |  |
| Tertiary | 6366 (29.4) | 73 (10.0) | 6439 (28.8) |  |
| Smoking status, n (%) |  |  |  | <0.001 |
| Never | 15384 (71.2) | 444 (60.8) | 15828 (70.8) |  |
| Current | 5957 (27.6) | 268 (36.7) | 6225 (27.9) |  |
| Former | 279 (1.3) | 18 (2.5) | 297 (1.3) |  |
| Drinking status, n (%) |  |  |  | <0.001 |
| Never | 17092 (79.1) | 492 (67.6) | 17584 (78.7) |  |
| Current | 4362 (20.2) | 226 (31.0) | 4588 (20.5) |  |
| Former | 159 (0.7) | 10 (1.4) | 169 (0.8) |  |
| Adequate physical activity, n (%) | 8568 (42.6) | 354 (53.7) | 8922 (42.9) | <0.001 |
| Diabetes, n (%) | 178 (0.6) | 17 (2.2) | 195 (0.6) | <0.001 |

*Abbreviations*: BMI, body mass index; DZ, dizygotic; SD, standard deviation; IQR, interquartile range.

Table 2. ORs (95% CIs) of overweight or obesity-hypertension association (normal BMI as the reference) from the GEE models

| Models | No. of cases | OR (95% CI) |
| --- | --- | --- |
| Model^a^ | 27977 | 2.03 (1.73,2.39) |
| Model^b^ | 20888 | 1.91 (1.62,2.25) |
| Model^c^ | 19394 | 1.94 (1.64,2.30) |

*Abbreviations*: BMI, body mass index; GEE, generalized estimating equation; OR, odds ratio; CI, confidence interval.

^a^Adjusted for age and sex.

^b^Adjusted for age, sex, marital status and education.

^c^Adjusted for age, sex, marital status, education, smoking status, alcohol consumption, physical activity and diabetes.

Table 3. ORs (95% CIs) for the association between overweight or obesity and hypertension in co-twin control analyses using hypertension discordant twin pairs from the conditional logistic regressions

| Co-twin without hypertension | Twin with hypertension | | | | | | | |
| --- | --- | --- | --- | --- | --- | --- | --- | --- |
|  | MZ+DZ | |  | DZ | |  | MZ | |
|  | Normal BMI | Overweight |  | Normal BMI | Overweight |  | Normal BMI | Overweight |
| Normal BMI | 175 | 77 |  | 72 | 56 |  | 103 | 21 |
| Overweight | 45 | 128 |  | 22 | 56 |  | 23 | 72 |
| OR (95% CI)^a^ | 1.63 (1.12,2.37) | |  | 2.39 (1.45,3.95) | |  | 0.91 (0.51,1.65) | |
| OR (95% CI)^b^ | 1.60 (1.09,2.33) | |  | 2.44 (1.46,4.09) | |  | 0.84 (0.45,1.54) | |
| OR (95% CI)^c^ | 1.80 (1.18,2.74) | |  | 2.86 (1.57,5.21) | |  | 0.89 (0.46,1.72) | |

*Abbreviations*: BMI, body mass index; MZ, monozygotic; DZ, dizygotic; OR, odds ratio; CI, confidence interval.

The 425 (206 DZ and 219 MZ) hypertension discordant pairs were divided into four groups with respect to exposure (overweight) status. In 175 (72 DZ and 103 MZ) twin pairs, both were normal BMI. In 128 (56 DZ and 72 MZ) twin pairs, both were overweight. In 77 (56 MZ and 21 MZ) twin pairs, the healthy (hypertension-free) co-twin had normal weight and the diseased twin was overweight. In 45 (22 DZ and 23 MZ) twin pairs, the diseased co-twin had normal BMI and the healthy twin was overweight.

^a^Adjusted for sex.

^b^Adjusted for sex, marital status, education.

^c^Adjusted for sex, marital status, education, smoking status, alcohol consumption, physical activity and diabetes.

Table 4. Differences in ORs (95% CIs) for the unmatched GEE models and matched co-twin control analyses (the difference in overweight or obesity between unmatched and co-twin matched controls)

| Models | MZ+DZ | | |  | DZ | | |  | MZ | | |
| --- | --- | --- | --- | --- | --- | --- | --- | --- | --- | --- | --- |
|  | No. of cases | OR (95% CI) | *P* value |  | No. of cases | OR (95% CI) | *P* value |  | No. of cases | OR (95% CI) | *P* value |
| Model^a^ | 27656 | 1.26 (1.03,1.55) | 0.023 |  | 27437 | 1.22 (0.91,1.63) | 0.190 |  | 27450 | 1.30 (0.99,1.72) | 0.062 |
| Model^a^ | 20574 | 1.32 (1.07,1.62) | 0.009 |  | 20369 | 1.26 (0.94,1.69) | 0.122 |  | 20375 | 1.36 (1.03,1.81) | 0.032 |
| Model^a^ | 19115 | 1.30 (1.05,1.62) | 0.016 |  | 18924 | 1.26 (0.92,1.72) | 0.146 |  | 18936 | 1.34 (1.00,1.79) | 0.052 |

*Abbreviations*: MZ, monozygotic; DZ, dizygotic; GEE, generalized estimating equation; OR, odds ratio; CI, confidence interval.

^a^Adjusted for age and sex.

^b^Adjusted for age, sex, marital status and education.

^c^Adjusted for age, sex, marital status, education, smoking status, alcohol consumption, physical activity and diabetes.

Table 5. Correlations (95% CIs) for BMI, hypertension, and BMI-hypertension by zygosity

| Zygosity | Within-trait, cross-twin | | Cross-trait,  within-twin | Cross-trait,  cross-twin |
| --- | --- | --- | --- | --- |
|  | BMI | Hypertension |  |  |
| MZ | 0.70 (0.68,0.72) | 0.73 (0.67,0.79) | 0.19 (0.09,0.24) | 0.18 (0.13,0.23) |
| DZ | 0.51 (0.48,0.53) | 0.57 (0.45,0.67) | 0.22 (0.16,0.28) | 0.10 (0.04,0.16) |

*Abbreviations*: BMI, body mass index; *MZ*, monozygotic*; DZ*, dizygotic; CI, confidence interval.

Table 6. Parameter estimates (95% CIs) from the best-fitting bivariate ACE full model of BMI and hypertension

|  | Variance components | |  | Correlation | |
| --- | --- | --- | --- | --- | --- |
|  | BMI | Hypertension |  |  |  |
|  |  |  |  | *r*_Ph_ | 0.21 (0.17,0.25) |
| A | 0.45 (0.41,0.49) | 0.32 (0.08,0.59) |  | *r*_A_ | 0.59 (0.44,1.00) |
| C | 0.36 (0.32,0.40) | 0.41 (0.15,0.63) |  | *r*_C_ | -0.06 (-1.00,1.00) |
| E | 0.19 (0.19,0.20) | 0.27 (0.21,0.33) |  | *r*_E_ | 0.05 (-0.05,0.14) |

*Abbreviations*: BMI, body mass index; CI, confidence interval; A, Additive genetic factors; C, shared environmental factors; E, non-shared environmental factors; *r*_Ph_, phenotypic correlation; *r*_A_, genetic correlation; *r*_C_, shared environmental correlation; *r*_E_, non-shared environmental correlation.
